# Dr. George W. Betts

**Published:** 1917-07

**Authors:** 


**Dr. George W. Betts,** N. Y. University, 1880, died at his home in Pulaski of apoplexy, April 18, aged 58.

